# Effect of Peanut Protein Treated with Alkaline Protease and Flavorzyme on BALB/c Mice

**DOI:** 10.3390/foods12132634

**Published:** 2023-07-07

**Authors:** Erlian Shu, Shuo Wang, Bing Niu, Qin Chen

**Affiliations:** School of Life Sciences, Shanghai University, Shanghai 200444, China; sxk0914@shu.edu.cn (E.S.); mia98@shu.edu.cn (S.W.); bingniu@shu.edu.cn (B.N.)

**Keywords:** peanut allergy, enzyme treatment, BALB/c mice, proinflammatory factor, IgE, intestinal flora

## Abstract

This article aims to analyze the effects of enzyme treatment concentration, temperature, and time on peanut protein so as to obtain an optimal enzymatic hydrolysis condition for flavorzyme (Fla) and alkaline protease (Alk). The results were as follows: enzymatic hydrolysis temperature 60 °C and 55 °C, enzyme concentration 10% and 4%, enzymatic hydrolysis time 80 min and 60 min, and double enzyme hydrolysis ratio 2% Fla + 5% Alk, respectively. The BALB/c mice were sensitized with gavage of peanut protein before and after enzyme treatment to evaluate the effects of different enzyme treatments on peanut allergenicity. Compared with the mice sensitized with raw peanuts, the weight growth rate of the mice sensitized with enzyme treatment peanut increased but not as much as the control, the degranulation degree of mast cell and basophils decreased, the inflammatory infiltration and congestion in jejunum and lung tissue decreased, the expression of proinflammatory factors and thymic stromal lymphopoietin (TSLP) gene decreased, and the secretion of specific antibodies (IgE, and IgG) decreased, and the binding ability of peanut protein with peanut-specific IgE antibodies decreased as well. The results above indicate that the allergenicity of peanut protein decreases after enzyme treatment and the dual enzyme (Fla + Alk) treatment can be much more efficient.

## 1. Introduction

Nowadays, over 170 foods can provoke allergenic reactions, but 90% of incidences are believed to be caused by allergenic proteins present in milk, egg, soy, crustacean/shellfish, fish, tree nut, peanut, and wheat [1,2]. Among the fatal cases of food allergies, 59% are accounted for by peanuts [3]. Peanut allergy can cause symptoms ranging from mild (rash, urticaria, itching, runny nose, edema, difficulty breathing, diarrhea, etc.) to life-threatening cases [4,5,6]. Currently, there are 17 allergens in peanuts, named Ara h 1–3, and Ara h 5–18 recognized by the WHO/IUIS allergen Nomenclature Subcommittee for peanut allergens (http://www.allergen.org/search.php?Species=Arachis%20hypogaea, accessed on 1 July 2023). The main allergens of peanuts include Ara h 1, Ara h 2, Ara h 3, and Ara h 6, which can be found in 90% of allergic patient serum, with Ara h 1 being the most abundant, accounting for 12–16% of total peanut protein [7].

During processing, food allergens undergo various physical and chemical reactions, leading to changes in the protein structure [8]. The current processing methods mainly include boiling, frying, roasting, microwave, ultrasonic treatment, and enzyme treatment. Existing studies have shown that boiled peanuts can reduce the solubility and sensitization by changing chemical structures [9,10]; with the increase in frying time, the solubility of peanut allergens and the binding capacity of IgE can be significantly reduced [11,12]; high pressure and microwave treatment can cause peanut allergens to form insoluble macromolecular protein polymers, thereby reducing the allergenicity of peanut allergens to varying degrees [10,13,14]; ultrasonic treatment of peanuts can lead to the formation of high-pressure and high-temperature environments between molecules, leading to the breaking of peptide bonds in peanut allergens, thereby reducing their allergenicity [6,15,16]; after roasting, peanut allergens can not only resist simulated gastric juice digestion more effectively, but also promote intestinal absorption and thus the allergenicity of these peanuts increase [11,17,18].

In addition to the above processing methods, enzyme treatment is one of the most promising methods for producing hypoallergenic peanuts, as it usually does not involve harmful substances [7]. Enzymatic treatment can reduce peanut allergy by degrading allergens and changing the structure of antigen determinants. It can also play a role by modifying allergens and reducing peanut allergy, including forming covalent bond in polypeptide chains and allergens, polymerizing allergens and blocking allergenic epitopes [19]. Nona et al. [20] found that enzyme treatment can effectively reduce the main allergens in raw peanuts and their ability to bind to IgE by treating raw peanuts with alcalase, papain, and cellulase. Cabanillas [21] found that 90 min of alkaline enzyme treatment can completely eliminate the IgE binding reactivity of peanut protein extract. Maleki et al. [22] effectively degraded Ara h 1 and Ara h 2 in roasted peanuts by treating them with α-chymotrypsin and trypsin.

Flavorzyme (Fla) and alkaline protease (Alk), evaluated in this study, are widely used in the food industry and are harmless to the human body [23,24,25], BALB/c mice were used to systematically evaluate the effects of enzyme treatment on peanut sensitization in terms of morphological changes and weight growth rate, basophil changes in peritoneal lavage fluid, histological sections of jejunum and lung, gene expression of TSLP, serum cytokines, and specific antibodies.

## 2. Materials Section

### Materials

Peanuts were purchased at Shanghai Walmart supermarket, flavorzyme and alkaline protease were both purchased from Shanghai Macklin Biochemical Co., Ltd. (Shanghai, China), and SPF grade BALB/c mice were purchased from Shanghai Jie Sijie laboratory animal Co., Ltd. (Shanghai, China).

BCA protein concentration assay reagents, skim milk, and TMB chromogentic reagent were purchased from Shanghai Huisheng biological company. Pre-stained protein markers containing from 11 kDa to 180 kDa were purchased from Shanghai Tiangong Technology. SDS-PAGE gel Kit (10%) was purchased from Shanghai Epizyme Biotechnology. Hanks’ balanced salt solution (1 × HBSS, phenol red free, and sterile) was purchased from Sigma, USA. Toluidine blue dye solution was purchased from Wuhan Good Biotechnology. The 4% paraformaldehyde fixative solution was purchased from Shanghai QCbio Science & Technologies Co. Ltd. Trizon reagent total RNA extraction reagent was purchased from Cowin Biotechnology. TB Green ^®^ Premix Ex Taq ™ was purchased from Takara, Japan. Mice IL-5, IL-6, and IFN-γ reagents were purchased from Shanghai Lianke company. HRP goat anti-rabbit IgG1 and HRP goat anti-rabbit IgG2a were purchased from Abclonal (Shanghai, China) Trading. HRP goat anti-rabbit IgE was purchased from Thermo Fisher Scientific (Shanghai, China). Other experimental reagents were purchased from Sinopharm Chemical Reagent.

## 3. Methods

### 3.1. Effect of Fla and Alk Treatment on the Protein Content of Peanut Protein

We weighed 10 g of peeled peanut, crushed them with a high-speed grinder, and added 100 mL Tris-HCl buffer solution (pH 8.0) at 4 °C for 30 min with constant stirring. Then, it was centrifuged at 4000× *g* for 30 min. Finally, the supernatant was collected and filtered through a 0.22 μM syringe filter to obtain soluble crude peanut protein by filtration and stored at −20 °C. Peanut protein content was treated with different temperatures, times, concentrations as well as different ratios of Fla and Alk, and the protein concentrations were determined before and after enzymatic hydrolysis using a BCA Kit. The optimized enzymatic conditions for Fla and Alk were as follows: 60 °C, 55 °C, 10% ± 4% enzyme concentration, 80 min, 60 min enzymatic hydrolysis time, and ratio of two enzyme hydrolysis: 2% Fla + 5% Alk (see Supplementary Materials).

### 3.2. Effects of Fla and Alk-Treated Peanut Protein in BALB/c Mice

#### 3.2.1. Protocol for Peanut Protein Sensitization of BALB/c Mice

Thirty 4-week-old female BALB/c mice were randomly divided into three groups: negative control (PBS), positive control (raw peanut) and experimental group (enzymes-treated peanut). The mice were adaptively fed for 7 days and their current body weight was recorded as W_1_, and then sensitized as follows: on days 1, 8, 15 and 23, the mice were gavaged with 0.5 mL PBS solution, 10 mg/mL raw peanut protein solution or enzyme-treated peanut protein solution. On the 30th day, mice were treated with 0.5 mL PBS solution, 50 mg/mL raw peanut protein solution or enzyme-digested peanut protein solution with gavage. After 30 min, the body weight was recorded as W_30_, and then the mice were sacrificed to collect the serum. Peritoneal lavage fluid, jejunum, small intestine and lung tissues were preserved in 4% paraformaldehyde fix solution for subsequent analyses.

#### 3.2.2. Effect of Fla and Alk-Treated Peanut Protein on Morphology and Weight Growth Rate in Mice

To observe the morphological changes of mice before and after sensitization and record the body weight of sensitized mice after 30 days in a normalized way, the mouse body weight growth rates was calculated with the following formula:

$$\mathrm{Weight}\;\mathrm{gain}\;\mathrm{rate}=\frac{\left(W_{30}-W_{1}\right)}{W_{1}}\times 100\%$$

#### 3.2.3. Effects of Fla and Alk-Treated Peanut Protein on Mice Basophils

At the time of dissection, 3 mL HBSS (1×) was injected into the abdominal cavity of the mice, and after gentle massage for 1 min, the solution was aspirated and 300 μL of it was applied onto glass slides until dry. After staining with toluidine blue for 2 min, we rinsed the slides until there was no staining solution, and then left to dry. Thereafter, the basophils in the peritoneal lavage fluid were observed under an optical microscope.

#### 3.2.4. Effects of Fla and Alk-Treated Peanut Protein on Intestine and Lung

Jejunum and lung tissues of mice were treated with xylene, embedded in paraffin and cut into 5 μM sections. Hematoxylin and eosin (H&E) staining was used to observe the lesions in the jejunum and lung tissues of mice under an optical microscope. The degree of inflammation was evaluated based on the infiltration of macrophage, peribronchiolar inflammatory cell infiltration and alveolar inflammatory cell. Alveolar stenosis and alveolar wall thickening were also indicators for inflammation evaluation.

#### 3.2.5. Effect of Fla and Alk-Treated Peanut Protein on Antibody Secretion

ELISA assays were performed with peanut-specific IgE, IgG1, and IgG2a antibodies. Serums with different dilution concentrations were used as primary antibodies (IgG1 dilution ratio 1:3000 and IgG2a dilution ratio 1:5000), and goat anti-mouse IgE/IgG1/IgG2a-HPR was used as secondary antibody (40,000 fold dilution for IgG1 and 5000 fold dilution for IgG2a). ELISA assays were performed at the same time and under the same conditions, with standard curves plotted. Coat 10 µg peanut protein were added to each well in 96-well plates and then coated for 14 h at 4 °C. The plates were rinsed three times with wash solution and 200 μL mice serum was added to each well at appropriate dilution and incubated at 37 °C for 2 h for primary antibody incubation. The plates were rinsed three times and 200 μL serum was added to each well of appropriate dilution of goat anti-mouse IgE/IgG1/IgG 2a-HPR and incubated at 37 °C for 1 h for secondary antibody incubation. The plates were rinsed three times and incubated with 200 μL TMB chromogentic reagent for 15 min in the dark at room temperature, and 50 μL 2 M sulfuric acid solution was added to terminate the reaction; OD_450_ for each well was measured using a microplate reader. Peanut-specific IgE, IgG1, and IgG2a antibodies were calculated according to the standard curve.

Indirect competitive ELISA: To evaluate the potential sensitization of raw peanut and enzymatically hydrolyzed peanut proteins, the binding capacity of raw peanut and enzymatically hydrolyzed peanut proteins to peanut-specific IgE was detected using indirect competitive ELISA. Raw peanuts and enzymatically hydrolyzed peanut proteins were used as inhibitors. Coat 10 µg peanut protein (200 μL) were added to each well in 96-well plates stored overnight at 4 °C. After rinsing the plates with washing solution containing 0.05% Tween, and blocking with 5% skim milk for 1 h, raw peanut and enzymatically peanut protein were diluted to 10^−3^, 10^−2^, 10^−1^, 10^0^, and 10^1^ μg/mL with mice serum (100,000-fold diluted) and incubated at 37 °C for 1 h. The blocked 96-well plates were again washed, and raw peanut and enzymatically digested peanut protein incubated with serum were added to each well at 200 μL, incubated at 37 °C for 2 h. After removing the washing solution, the 96-well plates were individually loaded with appropriate dilutions of goat anti-mouse IgE-HRP and incubated at 37 °C for 1 h. Then, in the 96-well plates, 200 μL TMB chromogentic reagent was added for 15 min, and an additional 50 μL 2M sulfuric acid was added to terminate the reaction, the meaning is retained. OD_450_ was detected using a microplate reader. The inhibition rate was calculated using the following formula:

$$Inhibition\;rate\left(\%\right)=\left(1-\frac{B}{B0}\right)\times 100$$

B and B0 are the absorbance values for wells with and without inhibitor, respectively.

#### 3.2.6. Effects of Fla and Alk-Treated Peanut Protein on the Secretion of Proinflammatory Factors and TSLP Gene Expression

Total RNA was extracted from small intestine and lung tissues using a kit. Purity (A260/A280 ratio) and RNA concentration were measured using a spectrophotometer, according to the manufacturer’s instructions. After the small intestine and lung tissues were extensively ground with liquid nitrogen, the total RNA of mice was extracted using an extraction kit. Mice total RNA was reverse transcribed to cDNA. cDNA was used as the template for real-time polymerase chain reaction (PCR) amplification and analysis of the TSLP gene was performed using real-time PCR with primers for the gene of interest (TSLP) or a housekeeping gene (GAPDH) (TSLP sequence is ACGGATGGGGCTAACTTACAA/AGTCCTCGATTTGCTCGAAC, and GAPDH sequence is CCAGGTTGTCTCCTGCGACTT/CCTGT TGCTGTAGCCG-TATTCA). The values of housekeeping genes were used as controls to normalize the results for the gene of interest. The Ct of each sample was calculated using Microsoft Office 2016 Excel software. The expression was calculated using the following formula:

$$2^{-\Delta \Delta Ct}=2^{-({\Delta Ct}_{expriment}-{\Delta Ct}_{control})}=2^{-[{\left({Ct}_{expriment}-{Ct}_{housekeeping}\right)}_{expriment}-{\left({Ct}_{control}-{Ct}_{housekeeping}\right)}_{control}]}$$

#### 3.2.7. Effects of Fla and Alk-Treated Peanut Protein on the Intestinal Flora of Mice

The intestinal content samples from mice were collected and total DNA was extracted. PCR was performed with forward (338f) and reverse (806r) primers targeting the v3-v4 region of 16sRNA from the bacteria of all the intestinal content samples. The PCR products were checked using the agarose gel electrophoresis; referring to the preliminary quantification results of electrophoresis, the products were further examined using fluorescence quantification, and finally high-throughput sequencing was performed using the Illumina Mi Seq platform. The sequence of 338f was 5′-ACTCCTACGGGAGGCAGCA-3′, and that of 806r was 5′-GGACTACHVGGG TWTCTAAT-3′. PCR reaction conditions were 95 °C for 3 min; and 27 cycles of 95 °C for 30 s, 55 °C for 30 s, 72 °C for 45 s and 72 °C 10 min. The concentration of agarose gel electrophoresis was 2% and the voltage was 5 V/cm for 30 min. The fluorometric detection employed the QuantiFluortm ST Blue Fluorescence quantitation system for quantitative detection. The normalized OTU table was used to calculate species phylogeny, richness (total number of OTUs) and the Shannon index. Hellinger corrected Bray–Curtis similarity matrices were used for cluster analysis, and ADONIS and ANOSIM were used to test differences between treatments.

### 3.3. Statistical Analysis

The statistical analysis of differences in data results was performed using *t*-test and one-way ANOVA with SPSS 20.0 software. Data are expressed as mean ± SD, and all experiments were repeated at least three times and plotted using GraphPad Prism 4 software, unless otherwise specified. In general, significance * *p* ≤ 0.05 was considered statistically significant.

## 4. Results

### 4.1. Effects of Fla and Alk-Treated Peanut Protein on Morphology and Weight Growth Rate in Mice

**Figure 1:**
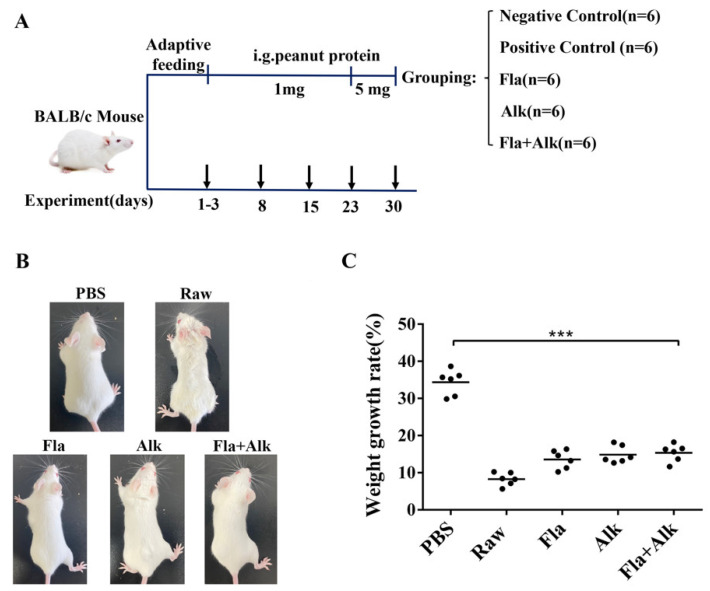
Sensitization flow chart of BALB/c mice (**A**) and effects of Fla and Alk-treated peanut on morphology (**B**) and weight growth rate of mice (**C**). (***) *p*-value < 0.001.

A flowchart of mice sensitization is shown below (Figure 1A). Thirty 4-week-old female BALB/c mice were randomly divided into five groups. Mice were sensitized at day 7 after being housed in an SPF-grade animal house.

After the sensitization cycle ended, the mice morphological changes before and after sensitization were observed and the weight growth rate were calculated. Compared with the PBS group, after peanut protein sensitization, the mice weighed less, their fur became sparse and yellow hair, scratching behavior and hair loss appeared, which were especially more obvious in the raw peanut group (Figure 1B). Figure 1C shows that the weight growth rate reached 35% in the control group and the rate was significantly lower in the peanut-sensitized mice than the control group, whereas after enzymatic treatment, the weight growth rate slightly increased and reached about 10% without significant differences among the three enzyme-treated mice. The decreased weight growth rate of the mice indicated that peanut sensitization might cause inappetence, slow weight growth, etc., which was effectively alleviated via the enzyme treatment.

### 4.2. Effects of Fla and Alk-Treated Peanut Protein on Mice Basophils

**Figure 2:**
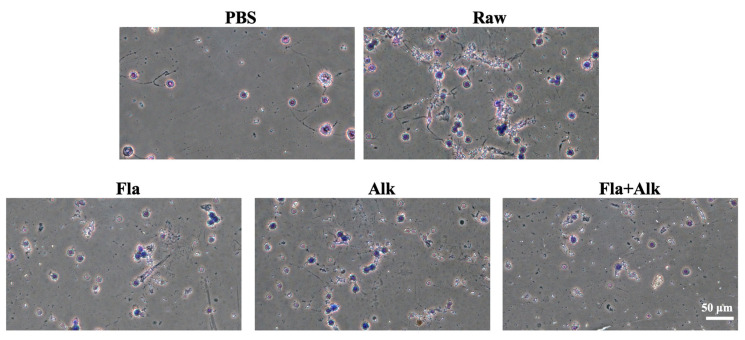
Effect of peanut treated with Fla and Alk on basophils.

The changes in each cell in the intestinal fluid were observed under an optical microscope, and the results are shown in Figure 2. Compared with the PBS group, peanut protein-sensitized mice, the extent of basophil aggregation was significantly increased, the cell was swelled or broken, the structure was loose, showing a diffuse state by week four, indicating that the degree of basophil degranulation was increased, thereby highlighting that peanut protein has strong sensitization effect on mice. After enzymatic treatment, cell degranulation decreased and cell aggregation improved.

### 4.3. Effects of Fla and Alk-Treated Peanut Protein on Intestine and Lung

**Figure 3:**
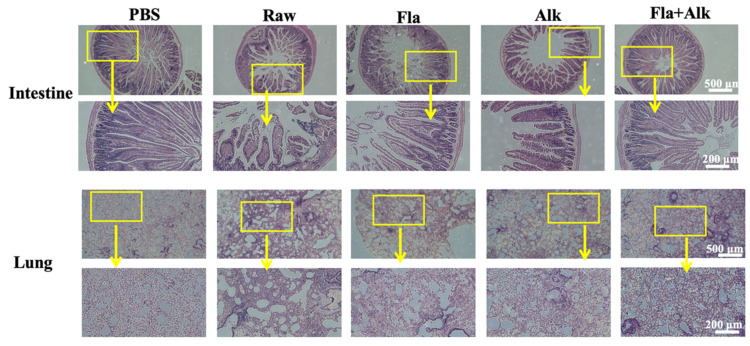
Effects of peanut treated with Fla and Alk on intestine and lung.

As shown in Figure 3, the intestinal tissue structure of mice in the PBS group was clear, the villi of the small intestine were arranged regularly, the bronchi were intact and arranged densely, and there was basically no inflammatory cell infiltration phenomenon. After peanut protein sensitization, the mice showed thickening of the intestinal lining, breakage of the small intestinal villi, severe bronchial breakage, and inflammatory cell infiltration. Compared with the mice in the raw peanut group, the intestinal villus arrangement tended to be regular, and phenomena, such as broken villi and broken bronchi in the small intestine, appeared significantly better in the enzyme-treated peanut group, indicating that the enzyme treatment effectively relieved the damage degree of peanut protein on the mice intestinal and lung tissues. Especially, the two enzyme treatment showed the best results.

### 4.4. Effects of Fla and Alk-Treated Peanut Protein on Antibody Secretion

**Figure 4:**
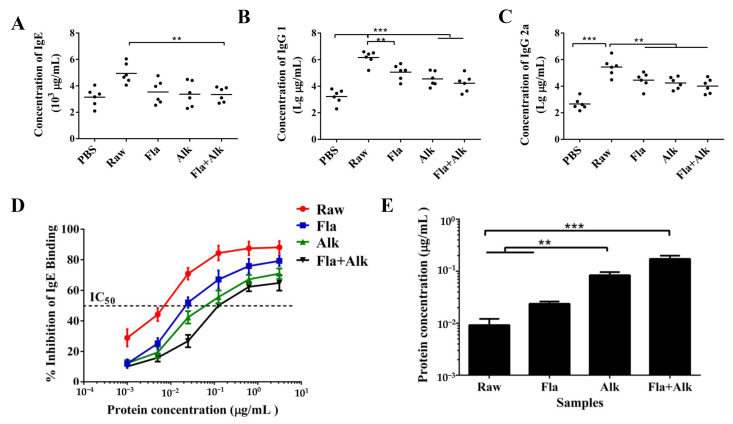
Influence of peanut treated with Fla and Alk on antibody secretion and antibody binding force. (**A**): concentration of IgE in serum, (**B**): concentration of IgG1 in serum, (**C**): concentration of IgG1 in serum, (**D**): IgE binding ability of peanut protein, (**E**): IC_50_ value of peanut protein binding to IgE. (**) *p*-value < 0.05, (***) *p*-value < 0.001.

The relative concentrations of specific antibodies, IgE, IgG1, and IgG2a, were determined, and the results are shown in Figure 4A–C. After peanut sensitization, the serum levels of immune cytokines IgE, IgG1 and IgG2a were significantly increased with IgG1/IgG2a > 1, indicating that the Th2-type immune response dominated in the peanut protein-sensitized mice. The amount of secreted antibody was significantly decreased in the serum of mice treated with the enzyme compared to the controls, indicating that the enzyme treatment effectively reduced protein sensitization.

The IgE binding capacity of peanut protein was evaluated, as shown in Figure 4D,E. The results showed that the binding rate of peanut protein to IgE decreased with increasing concentrations, and the inhibition rate of IgE binding to raw peanut group was higher than that of the enzyme-treated peanut at the same protein concentration. Compared to raw peanut protein, when peanut was treated with Fla, Alk, as well as Fla + Alk, the IC50 values increased from 0.0092 μg/mL to 0.0237, 0.0836, and 0.172 µg/mL, respectively. The results showed that the binding of peanut protein to specific IgE was significantly affected after the enzyme treatment. After the enzymatic treatment, the capacity of peanut protein to bind IgE was reduced.

### 4.5. Effects of Fla and Alk-Treated Peanut Protein on the Secretion of Proinflammatory Factors and TSLP Gene Expression

**Figure 5:**
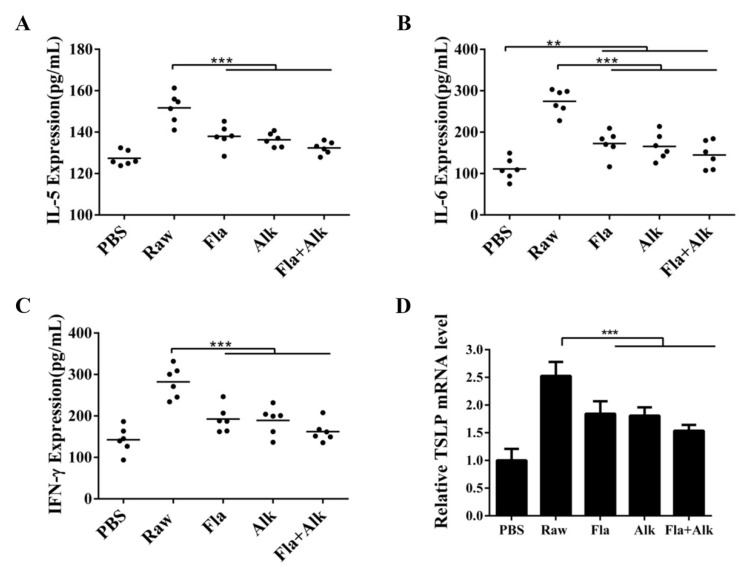
Effects of peanut treated with Fla and Alk on secretion of IL-5 (**A**), IL-6 (**B**), IFN-γ (**C**) and expression of TSLP gene (**D**). (**) *p*-value < 0.05, (***) *p*-value < 0.001.

Serum levels of pro-inflammatory cytokines IL-5, IL-6 and IFN-γ, and the results are shown in Figure 5A–C. Serum levels of inflammatory factors (IL-5, IL-6, and IFN-γ) in the groups of peanut-sensitized mice contents were all significantly higher than those of the control mice. Compared with the peanut-producing mice, the secretion of inflammatory factors in the enzyme-treated mice all decreased significantly, and there was no significant difference among the three enzyme-treated mice groups. These results suggest that there is a strong sensitization of mice to raw peanuts, leading to the production of inflammation and secretion of proinflammatory factors.

The TSLP expression in the lung tissues of mice in different treatment groups was compared. As shown in Figure 5D, after sensitization with raw peanut protein, the TSLP gene expression in mice significantly increased to 2.5-fold of that in mice in the PBS group, which was effectively inhibited by the enzyme treatment. After the enzymatic treatment, TSLP expression decreased significantly in the Fla + Alk-treated mice by 40%. These results indicate that raw peanut protein readily causes an increase in TSLP gene expression in mice, whereas enzyme treatment effectively reduces gene expression and subsequently suppresses peanut allergic responses.

### 4.6. Effects of Fla and Alk-Treated Peanut Protein on the Intestinal Flora of Mice

**Figure 6:**
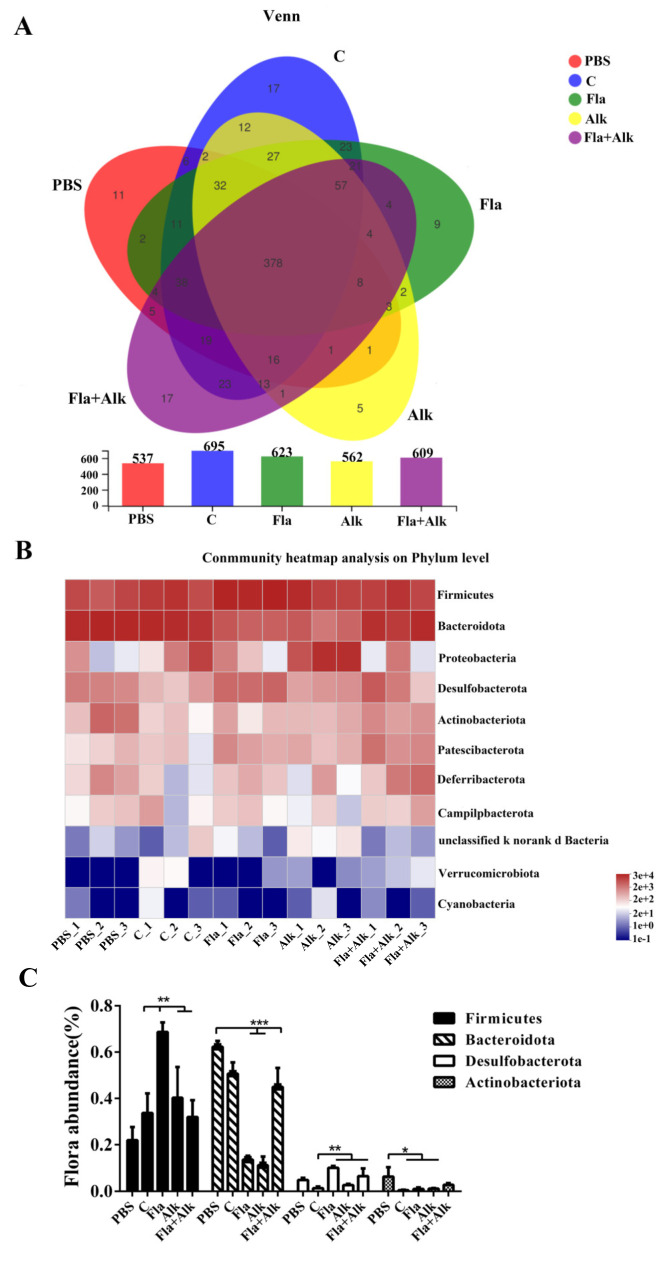
Effects of peanut treated with Fla and Alk on intestinal microflora diversity (**A**) and abundance (**B**,**C**) in mice. (*) *p*-value < 0.1, (**) *p*-value < 0.05, (***) *p*-value < 0.001.

The diversity of the intestinal flora in mice was analyzed, and the results are shown in the following figure. As shown in Figure 6A, the flora classes of the mice in the different treatment groups differed greatly, with 378 species common to the mice in the five treatment groups and 11, 17, 9, 5, and 17 species unique to the control, peanut grown, Fla, Alk, and Fla + Alk-treated mice, respectively. The mouse flora class was counted to obtain a plot of colony diversity. It is known from the figure that the diversity of the flora of mice increased in the peanut-sensitized group compared to the control group, and this phenomenon was reduced after the enzyme treatment. This result illustrated that raw peanut gavage caused gastrointestinal disorder in mice and they developed allergic phenomenon, while the allergenicity of peanut was effectively reduced after the enzyme treatment. The mice intestinal colony composition was further quantitatively analyzed, and the results are listed in Figure 6B. The results showed that the content of flora differed greatly between the mice in different treatment groups. A quantitative analysis of the mouse intestinal flora is presented in Figure 6C. It can be seen that the number of Bacteroidetes, Firmicutes, Desulfovibrio, and actinobacteria decreased in the enzyme-treated group compared to the peanut-sensitized group.

## 5. Discussion

Peanut allergy is an IgE-mediated immune response triggered by the consumption of peanut or foods containing peanut products, among others, leading to an allergic reactions, either in the digestive system or systemically [26]. The material basis of peanut allergy is an immunologically active peanut allergen protein [27]. Different processing methods may also lead to different degrees of structural changes in peanut protein and have different effects on the allergenicity of peanut protein, such as heat drying, frying, and roasting [28,29]. Studies have shown strong sensitization to peanut itself, whereas common peanut products are usually heat-treated and their sensitization is elevated after heat treatment [30]. Therefore, to reduce peanut sensitization, peanut is usually treated with an enzymatic solution. Enzyme crosslinking is a new method of modern food processing, wherein peanut-sensitized protein becomes a small peptide segment under the decomposition of the enzyme system, and the protein undergoes structural changes during the process of crosslinking and aggregation, destroying the original protein epitopes or generating new epitopes, which, in turn, has an impact on peanut protein sensitization [31]. Zheng et al. [32] have showed that flavor protease and alkaline protease were the most effective proteases in the process of hydrolyzing defatted peanut protein powder, and they also played a significant role in hydrolyzing peanut-purified protein. Shi et al. [33] treated crude and purified peanuts proteins with alkaline protease, neutral enzyme, papain and flavor protease, respectively, to analyze the protein solubility, subunit and conformation and amino acid composition changes of residues after enzymatic hydrolysis, and compared their enzymatic efficiency, and showed that both crude and purified proteins could be hydrolyzed by these four enzymes, among which alkaline protease and flavor protease were the most effective in hydrolyzing allergens. During the enzymatic hydrolysis of the two proteins, the effect was similar after treatment with alkaline protease, while the enzymatic hydrolysis of the purified protein was less effective when flavor protease was used than that of the peanut crude protein. Therefore, flavor protease and alkaline protease were used to treat peanut protein in this paper, both of which are food grade enzymes and are not toxic and harmless to humans. Furthermore, in addition to the commonly used single enzyme treatment, this paper tried to jointly use two enzymes to enzymatically hydrolyze peanut.

In our results, the protein concentration extracted from peanut after treatment with different enzymatic hydrolysis conditions was consistent with the SDS PAGE results, and both Fla and Alk could reduce the concentration of peanut allergen to various degrees and degrade major peanut allergens (Ara h 1, Ara h 2, Ara h 3, etc.) into small molecular weight peptides; they have the most obvious effect on Ara h 1 degradation, which is speculated to cause peptide degradation or structural changes during enzymatic hydrolysis. It is well known that the enzymatic hydrolysis efficiency is closely related to the enzyme concentration, time, and temperature conditions. Therefore, in this paper, the enzyme treatment time, enzyme concentration and temperature were screened to investigate the effects of different conditions on the physicochemical properties and biological activity of peanut protein. The results showed that the protein concentration decreased with the increase in enzyme treatment time and enzyme concentration, and the decreasing effect was no longer obvious after a certain threshold was reached. For the temperature screening, the protein concentration with the increase in temperature first decreased and then increased, and the degradation effect reached an optimum at the optimum temperature of the enzyme. The optimal enzymatic conditions for Fla and Alk hydrolysis were as follows: 60 °C, 55 °C, enzyme concentration of 10% and 4%, enzyme hydrolysis time of 80 min and 60 min, respectively, and the two enzyme hydrolysis ratio was as follows: 2% of Fla + 5% of Alk.

Liu [34] tested BALB/c mice with different allergenic proteins and found that BALB/c mice could well distinguish the allergenicity of different proteins, hence, scholars mostly use BALB/c mice as a food allergenicity evaluation model. In this paper, BALB/c mice were gavaged with peanut protein treated with different enzymes, and various allergic indicators of the mice were observed and detected. Zhang et al. [35] observed pathological sections of the jejunum while establishing a peanut allergy model and found that anaphylaxis can lead to lesions in the jejunum injury. In this paper, we investigated the pathological parameters of the mice and found that the peanut-sensitized mice group were morphologically thin, their hair turned sparse and yellow, and their body weight growth rate significantly decreased. Intraperitoneal lavage fluid smear observations indicated that enzymatic treatment reduced the degree of degranulation of basophils compared to the peanut-sensitized group. The villi arrangement in the small intestine of mice tended to be regular, and the phenomena of villi and bronchi in the small intestine were significantly alleviated, indicating that the damage caused by peanut protein to the intestine and lung tissues of mice was effectively alleviated after the enzyme treatment.

In addition, the secretion of various proinflammatory factors is induced after the mice develop peanut anaphylaxis. To investigate the effect of the peanut allergenic protein Ara h 2 on mouse sensitization, Zhang et al. [36] sensitized BALB/c mice with Ara h 2 protein treated with different processing methods and detected specific IgE, histamine, and cytokines (IL-4, IL-5, IL-13, and IFN-γ) in mice serum variation in content. The results showed that some indicators, such as cytokines were up-regulated in the serum of mice after protein sensitization, and Ara h 2 treated in different ways could trigger allergic reactions to different degrees in mice. Our serological results showed that after peanut sensitization, there were significant increases in the proinflammatory cytokines IL-5, IL-6, and IFN-γ content and TSLP gene expression, which is an important initiator of allergic responses. These results indicated that the mice developed immune responses. However, the secretion of proinflammatory factors and TSLP gene expression in mice treated with enzyme-treated peanut significantly decreased, indicating that enzyme treatment can effectively ameliorate the phenomenon of peanut allergy [37]. Based on the detection of mouse-specific antibody IgE, IgG1, and IgG2a contents, it was discovered that the levels of specific antibodies (IgE, and IgG) in the serum of mice in the enzyme-treated peanut group were significantly decreased compared with those in the peanut-sensitized group. The IgG1/IgG2a ratio was more than 1, indicating that the enzyme treatment could reduce the Th2-type allergic responses caused by peanut protein. Consistent with this finding, Xue et al. [38] revealed the effects of alkaline protease-treated gliadin on sensitization in mice, and detected serum IgE, histamine, and serum cytokine expression levels. They confirmed that alkaline protease hydrolysis can effectively reduce gliadin hypersensitivity by altering the protein structure. The above results confirmed the lower sensitization to enzyme-treated peanut and lower degranulation by basophils which revealed the cause of lower degree of breakage and lesions in the jejunum and lungs of mice.

In addition to the above common indicators, the intestinal flora has become an important research topic in allergy studies in recent years [39]. Intestinal Treg cells are closely associated with body immunity, and their differentiation is influenced by microbiota and their metabolites [40]. Treg cells in food allergic patients are particularly well differentiated and positively correlate with the microbiota in the gastrointestinal tract [41]. Early microbial colonization is characterized by dynamic changes in microbial diversity that reach equilibrium after the first few years of life and thereafter remain relatively stable without disturbance. Food allergy has been shown to induce alterations in the intestinal flora. Björkstén et al. [42] assessed changes in fecal bacterial composition within the first few years of life in infants with food allergy and showed that food allergic patients exhibit early onset symptoms of intestinal flora dysbiosis. Cahenzli et al. [43] showed that germ-free mice are more susceptible to develop allergic reactions due to the lack of intestinal flora, which shifts the Th1/Th2 balance toward Th2. Commonly used research indexes of intestinal flora are OTU taxa, diversity analysis of flora, community composition differentiation, and so on. In this paper, we analyzed the intestinal flora and showed that the diversity of intestinal flora was increased in peanut-sensitized mice, indicating that after peanut sensitization, the intestinal and the homeostasis were disrupted, which can be effectively alleviated after the enzyme treatment, further demonstrating that enzyme treatment can reduce peanut sensitization.

## 6. Conclusions

Treatment of peanut crude protein by Fla and Alk resulted in the following optimal enzymatic conditions: 60 °C, 55 °C, enzyme concentration of 10% to 4%, enzyme hydrolysis time of 80 min to 60 min, and double enzymatic hydrolysis ratio: 2% of Fla + 5% of Alk. Raw peanut protein was more allergenic to BALB/c mice, but the sensitization of the protein was decreased after Fla and Alk treatment, and the effect of the decreasing sensitization after different enzyme treatments was Fla + Alk > Alk > Fla.

From the experiments, we can see that peanut protein after enzymatic treatment can reduce peanut allergenicity and affect the intestinal flora of mice to a certain extent. However, the findings are far from enough. In future, we intend to observe the structural changes which lead to the reduction in peanut protein allergenicity by studying the diversification in the secondary and tertiary structures before and after the enzymatic hydrolysis of peanut protein. In addition, cell experiments can also be used to verify the mechanism of reducing the allergenicity of peanut protein treated with enzymatic hydrolysis in the intestine. Reducing the allergenicity of peanut protein by combining enzymatic hydrolysis and different processing methods is also a potential research direction.

## Data Availability

The data used to support the findings of this study can be made available by the corresponding author upon request.

## Supplementary Materials

The following supporting information can be downloaded at: https://www.mdpi.com/article/10.3390/foods12132634/s1, Table S1: Effects of Fla and Alk treatments on peanut crude protein content with different concentrations; Table S2: Effects of Fla and Alk treatments on peanut crude protein content at different times; Table S3: Effects of Fla and Alk treatments on peanut crude protein content at different temperatures; Table S4: Influence of Fla and Alk ratio on peanut crude protein content; Figure S1: Effects of Fla and Alk treatments on peanut crude protein content with different concentrations; Figure S2: Effects of Fla and Alk treatments on peanut crude protein content at different times; Figure S3: Influence of Fla and Alk treatments on peanut crude protein content at different temperatures; Figure S4: Influence of Fla and Alk ratio on peanut crude protein content.
