# Supplementary material for: Effect of Peanut Protein Treated with Alkaline Protease and Flavorzyme on BALB/c Mice
Source: Foods. 2023 Jul 7;12(13):2634. doi: 10.3390/foods12132634 (PMC10340410; doi:10.3390/foods12132634)
Supplement: Supplementary file 1 [file foods-12-02634-s001.zip › foods-2448578-supplementary.pdf]

Effects of Different Concentrations of Fla and Alk on the Content of Extracted Peanut Protein

After treating peanut crude protein with different concentrations of Fla and Alk, the protein concentration was determined and analyzed by SDS-PAGE. The results are shown below. From **Table S1**, it can be seen that the concentration of peanut crude protein decreases with the increase of Fla concentration, and there is no significant difference between different concentration treatment groups. When the Fla concentration was 2%, the protein concentration decreased by 29.5% compared to the control group. When the concentration of Fla reaches 10%, there is no significant decrease in the concentration of peanut crude protein. When the Alk concentration reaches 4%, there is no significant decrease in the concentration of peanut crude protein. At the same enzyme concentration, Alk has a stronger ability to reduce crude protein concentration than Fla, with a protein concentration reduction rate almost twice that of Fla.

Table S1 Effects of Fla and Alk treatments on peanut crude protein content with different concentrations

Fla Concent r-ation	Protein Concentration（μg/μL）	rate of decline	Alk Concentr-ation	Protein Concentration（μg/μL）	rate of decline
0	5.54±0.24 [a]	—	0	6.39±0.21[a]	—
2%	3.91±0.21 [b]	29.50%	1%	3.19±0.14[b]	50.10%
4%	3.77±0.12 [b]	32.00%	1.5%	3.08±0.21[b]	51.80%
6%	3.75±0.33 [b]	32.30%	2%	2.95±0.16[b]	53.80%
8%	3.69±0.08 [b]	33.30%	2.5%	2.82 ±0.07[b]	55.90%
**10%**	**3.65±0.19 [b]**	**34.10%**	3%	2.63±0.13[b]	58.80%
12%	3.64±0.05 [b]	34.20%	3.5%	2.49±0.09[c]	61.00%
			**4%**	**2.26±0.17[d]**	**64.60%**
			4.5%	2.25±0.05[d]	64.80%
			5%	2.25±0.09[d]	64.80%

SDS-PAGE electrophoresis showed that the bands of the main allergens in peanuts gradually became thinner and shallower after enzyme treatment. With the increase of enzyme concentration, the concentration of peanut protein decreased, and Ara h 1, Ara h 2, and Ara h 3 were degraded into small molecular weight peptide segments. Based on the comprehensive chart results, subsequent experiments were conducted using Fla with a concentration of 10% and Alk with a concentration of 4%.

A B

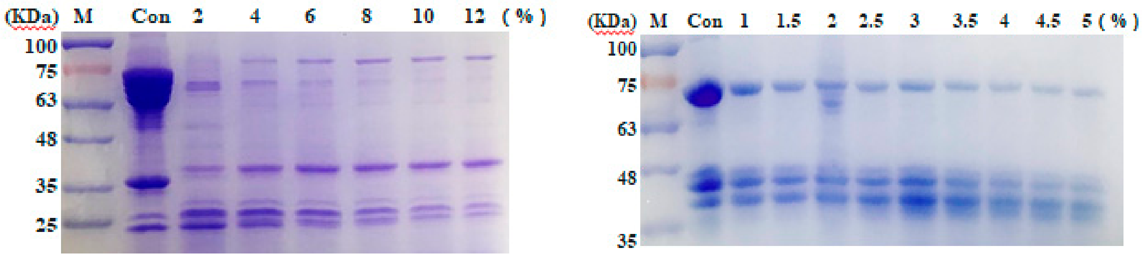

Figure S1 Effects of Fla and Alk treatments on peanut crude protein content with different concentrations

Note: A and B represent the crude protein content of peanuts treated with different concentrations of Fla and Alk, respectively; M is the standard protein Marker; Con is the control group of raw peanuts.

Effects of Fla and Alk treatments on peanut crude protein content at different times

After treating peanut protein with 10% Fla and 4% Alk at different concentrations for different times, the protein concentration was measured and analyzed by SDS-PAGE. The results are as follows. From the table, it can be seen that as the enzyme treatment time increases, the protein concentration significantly decreases. At the same treatment time, the protein concentration decrease rate after Alk treatment is higher than that of Fla, indicating that Alk has a stronger degradation effect on protein. After Fla enzymatic hydrolysis for 80 minutes, there was no significant decrease in crude protein content, while after Alk treatment for 60 minutes, there was no significant decrease in crude protein content.

Table S2 Effects of Fla and Alk treatments on peanut crude protein content at different times

Fla Processing Time (min)	Protein Concentration (μg/μL)	rate of decline	Alk Processing Time (min)	Protein Concentration (μg/μL)	rate of decline
0	5.33 ± 0.077^{a}	——	0	6.08 ± 0.33^{a}	——
20	3.24 ± 0.27^{b}	39.20%	20	2.38 ± 0.22^{b}	60.90%
40	3.01 ± 0.19^{b}	43.50%	40	2.21 ± 0.16^{c}	63.70%
60	2.96 ± 0.29^{b}	44.50%	**60**	$\mathbf{1.91\pm0.067^{d}}$	**68.60%**
**80**	$\mathbf{2.88\pm0.17^{b}}$	**46.00%**	80	1.90 ± 0.052^{d}	68.70%
100	2.73 ± 0.07^{c}	48.80%	100	1.88 ± 0.022^{d}	69.10%
120	2.69 ± 0.07^{c}	49.50%	120	1.86 ± 0.070^{d}	69.40%

The electrophoresis results showed that the content of peanut protein decreased with increasing enzymatic hydrolysis time. As shown in **Figure S2A**, the content of major peanut allergens such as Ara h 1 and Ara h 3 significantly decreased. After 80 minutes of enzymatic treatment, the protein no longer showed significant degradation. In view of this, the subsequent experiments used Fla and Alk with processing times of 80 and 60 minutes, respectively.

A B

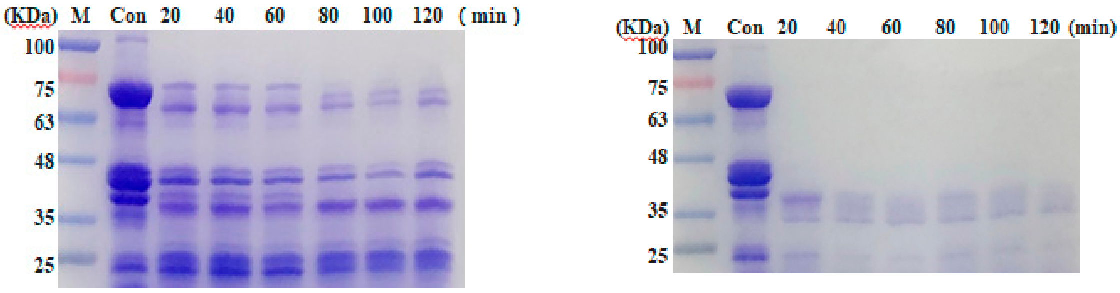

Figure S2 Effects of Fla and Alk treatments on peanut crude protein content at different times

Note: A and B represent the crude protein content of peanuts treated with Fla and Alk for

different times, respectively; M is the standard protein Marker; Con is the control group of raw peanuts.

Effects of Fla and Alk treatments on peanut crude protein content at different temperatures

After determining the enzyme concentration and processing time, peanut crude protein was treated with Fla and Alk at different temperatures. The results of protein concentration and protein electrophoresis are as follows. The protein concentration results show that after adding Fla and Alk, the protein concentration first decreases and then increases with the increase of temperature. Among them, the optimal temperature for Fla is 60 °C, and the optimal temperature for Alk is 55 °C.

Table S3 Effects of Fla and Alk treatments on peanut crude protein content at different temperatures

Fla Processing Temperature (°C)	Protein Concentration (μg/μL)	rate of decline	Alk Processing Temperature (°C)	Protein Concentration (μg/μL)	rate of decline
Con	4.37±0.23[a]	——	Con	5.43±0.24 [a]	——
50	2.35±0.11[c]	46.20%	45	1.98±0.15 [c]	63.50%
55	2.26±0.17[c]	48.30%	50	1.70±0.02 [c]	68.70%
**60**	**2.02±0.062[c]**	**53.80%**	**55**	**1.60±0.10 [c]**	**70.50%**
65	2.43±0.051[b]	44.40%	60	1.91±0.18 [c]	64.80%
70	2.67±0.076[b]	38.90%	65	2.18±0.11 [b]	59.90%
			70	2.46±0.14 [b]	54.70%

The electrophoresis results showed that the protein was degraded into small molecular weight bands, and the degradation effect of the main allergen was significant. The protein concentration showed a trend of first decreasing and then increasing with the increase of temperature. Therefore, for subsequent experiments, Fla and Alk were selected with temperatures of 60 °C and 55 °C, respectively。

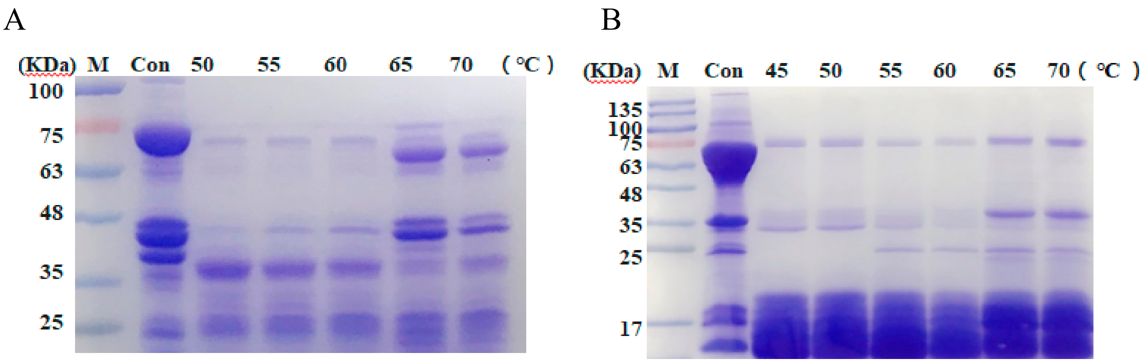

Figure S3 Influence of Fla and Alk treatments on peanut crude protein content at different temperatures

Note: A and B represent the crude protein content of peanuts treated with Fla and Alk at different temperatures, respectively; M is the standard protein Marker; Con as raw peanut control group.

Influence of Fla and Alk ratio on peanut crude protein content

Peanut crude protein was treated with different proportions of Fla and Alk, and the protein concentration was measured for SDS-PAGE analysis. The results are shown below. After the addition of enzymes, the concentration of peanut crude protein significantly decreased ($P<0.05$), and the addition of 2% Fla+5% Alk showed better enzymatic hydrolysis. The rate of protein decrease was not related to the order of enzyme addition.

Table S4 Influence of Fla and Alk ratio on peanut crude protein content

**Group**	**Protein Concentration (μg/μL)**	**rate of decline**	**Group**	**Protein Concentration (μg/μL)**	**rate of decline**
Con	2.85±0.12^{a}	——	10%B	1.88±0.06^{b}	34.0%
4%A	1.55±0.05^{b}	45.6%	5%B+1%A	1.29±0.06^{b}	54.7%
**2%A+2.5%B**	**1.36±0.01^{b}**	**52.3%**	5%B+1.3%A	1.28±0.10^{b}	55.1%
2%A+3.3%B	1.30±0.04^{b}	54.4%	**5%B+2%A**	**1.16±0.02^{b}**	**59.3%**
2%A+5%B	1.16±0.08^{b}	59.3%			

Note: A represents alkaline protease, B represents flavor protease; The protein decrease rate was all compared to that of raw peanuts, P<0.05.

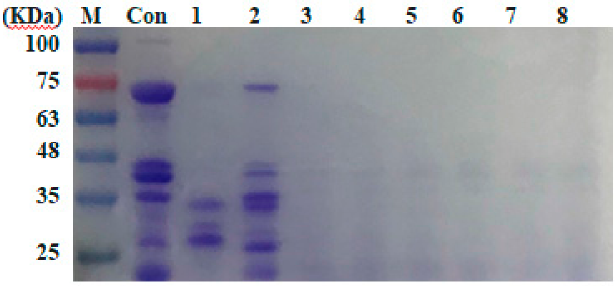

Figure S4 Influence of Fla and Alk ratio on peanut crude protein content

Note: Con represents raw peanuts, 1 represents the 4% A treatment group, 2 represents the 10% B treatment group, and 3-8 represents 2% A+2.5% B, 2% A+3.3% B, 2% A+5% B, 5% B+1% A, 5% B+1.3% A, 5% B+2% A treatment group.
